# Late Effects of Organ Preservation Treatment on Swallowing and Voice; Presentation, Assessment, and Screening

**DOI:** 10.3389/fonc.2019.00401

**Published:** 2019-05-21

**Authors:** J. M. Patterson

**Affiliations:** ^1^Institute for Health and Society, Newcastle University, Newcastle upon Tyne, United Kingdom; ^2^Speech and Language Therapy Department, City Hospitals Sunderland Foundation Trust, Sunderland, United Kingdom

**Keywords:** head and neck cancer, late radiation, function, dysphagia, aspiration, voice, assessment, screening

## Abstract

The prevalence of head and neck cancer (HNC) survivors is on the rise. Treatments for HNC can have a major deleterious impact on functions such as swallowing and voice. Poor functional outcomes are strongly correlated with distress, low quality of life, difficulties returning to work and socializing. Furthermore, dysphagia can have serious medical consequences such as malnutrition, dehydration, and pneumonia. A conservative estimate of the percentage of survivors living with dysphagia in the long-term is between 50 and 60%. Evidence is emerging that functions can worsen over time, sometimes several years following treatment due to radiation-associated fibrosis, neuropathy, intractable edema, and atrophy. Muscles lose their strength, pliability, stamina, and range, speed, precision, and initiation of movements necessary for swallowing and voice functions. Late treatment effects can go unrecognized, and may only be identified when there is a medical complication such as hospitalization for aspiration pneumonia. In the routine healthcare setting methods of evaluation include a detailed case history, a thorough clinical examination and instrumental assessments. Interventions for late treatment effects are limited and it is imperative that patients at risk are identified as early as possible. This paper considers the role of screening tests in monitoring swallowing and detecting aspiration in the long-term. Further work is indicated for addressing this pressing and increasingly common clinical problem.

## Introduction

The prevalence of head and neck cancer (HNC) survivors is on the increase, likely due to improvements in diagnostic technologies, treatment techniques, and a rising number of HPV-related oropharyngeal cancers, with patients presenting at a younger age with good survival outcome (1). HNC treatments can result in a multitude of side effects resulting in poor function. Post-treatment swallowing and voice difficulties are strongly related to psychosocial problems, poorer quality of life, anxiety, and low mood (2, 3). In addition, dysphagia can pose a serious medical threat, being associated with malnutrition, dehydration, and possibly pneumonia. Survivors may therefore live for a long time with significant symptom burden, which can increase in severity over time. This paper reports on the presentation, assessment, and potential screening tests for late treatment effects on swallowing and voice.

## The Complexity of Swallowing and Voice

Swallowing and voice are highly coordinated, specialized functions involving over 25 pairs of muscles under both voluntary and involuntary control. Swallowing is a finely tuned process, primarily because the oropharynx is a shared passageway for swallowing and respiration. It needs to be executed safely, avoiding spillage into the airway, and efficiently, to ensure adequate nourishment and hydration. Voice is produced by air passing through the vocal folds, causing the edges to vibrate rapidly. This sound is amplified by resonating cavities in the vocal tract, giving it a distinctive quality. The active articulators (tongue, lips and soft palate) modify the voiced sound into speech via quick and precise movements.

## Late Treatment Effects on Function

Surgical treatment for HNC may result in functional impairment, dependent on factors such as tumor site, volume resected, reconstruction, and the subsequent level of edema, scarring, and atrophy (4). Radiotherapy can create significant dysfunction both acutely and progressively also termed “late effects.” Acute radiotherapy side effects usually include pain, mucosititis, edema, and xerostomia. For some, this results in mild or short-term dysphagia and dysphonia, resolving by 3 months. This group are likely to return to their normal diet with minor limitations. This is a typical pattern for those treated by low dose radiotherapy (2, 5), but may be achieved by a small percentage of patients with advanced disease treated by intensity-modulated radiotherapy (IMRT) (6).

## Dysphagia

Evidence relating to the long-term impact of treatment on swallowing is emerging, with a number of potential patterns described by Christianen (5). Some patients experience progressive deterioration in the months and years following treatment, whereas others may have a sudden onset of severe dysphagia. Causes of these differing trajectories are not well-understood. Chronic edema and increased fibroblast formation are responsible for the overproduction of collagen, leading to scarring, and fibrosis (7). Consequently, tissues lose their elasticity and movements for adequate functioning are restricted. Atrophy can alter oropharyngeal anatomical relationships and reduce muscle strength. Furthermore, sensory impairment can blunt the cough response, should food, or drink enter the airway. Lower cranial nerve palsies (CN IX, X, and XII) have been noted, probably due to compression or direct nerve damage (8). Mandible osteoradionecrosis is a further late complication, resulting in exposed, non-healing bone (9). This is strongly associated with severe, chronic dysphagia (OR: 4.6, 95% CI: 2.1–10.3), making chewing painful and swallowing less efficient (10). Dysphagia is strongly associated with psychological distress, poorer quality of life, and is a top priority concern for HNC survivors (2, 11, 12)

### Prevalence of Late Effects Dysphagia

Little information is published on swallowing outcomes beyond 2 years. In our longitudinal study (*n* = 146 recruited pre-treatment), aspiration rates remained stable between one and 6 years following chemoradiotherapy (22–25%), with one half of these patients having silent aspiration (13). At 1 year, two patients had a laryngectomy for a dysfunctional larynx and four had a tracheostomy due to airway compromise. By 6 years, five further patients had a laryngectomy for a dysfunctional larynx (13). An Australian study reported a decline in swallowing efficiency over the same time frame (14). The RTOG 91–11 5 year follow up identified severe dysphagia in a third of the retained sample (15). SEER data reported a 49% rate of persistent dysphagia (16, 17), with older patients following non-surgical treatment being most at risk (18, 19).

### Dysphagia Associated Risk

Aspiration is a medical concern as it can lead to repeated chest infections, poor pulmonary function, and life-threatening aspiration pneumonia (20). In our 6 year follow up cohort, 28% reported at least one chest infection, with an increased risk ratio of 6.25 [*p* = 0.03 CI 1.1–35.7] for aspirators (13). Seven percent of those initially diagnosed with oropharyngeal cancer had a non-functioning larynx, requiring complex, and major reconstructive surgery (13). SEER data suggests the incidence of aspiration pneumonia in HNC is 8.7% (95% CI: 8.2–9.1) (17) and it is estimated that over 80% require hospital admission, half of whom transfer to intensive care (20). Case series and cross-sectional data report alarmingly high rates of aspiration-related deaths (21–23). For example, the 30-day mortality rate in a series of nasopharyngeal cancer patients was 51% (24). It is crucial that we identify those at risk of developing aspiration pneumonia as early as possible.

## Dysphonia

The prevalence of late voice problems are not well-documented. Small studies suggest deterioration in patient reports and clinical tests of voice up to 7 years post-treatment (25, 26). However, patients treated with IMRT appear to have better outcomes than those treated with conventional radiotherapy, in the long-term (3). A small percentage of patients may require a tracheostomy or laryngectomy, significantly altering voice quality (13).

## Assessment

### Clinical Swallowing and Voice Assessment

In the first instance, patients presenting with new symptoms, or deterioration in function should have recurrent or primary disease excluded as a possible cause. A comprehensive clinical history of late effects will include details on patients' perceptions of the onset, trajectory, and symptoms. Clinical information such as co-morbidities, reflux, chest status, oral intake, oral hygiene, and weight should be gathered. For older survivors, expected, age-related functional decline i.e., loss of muscle mass and elasticity, reduced saliva production should be taken into account. An examination will typically include and oral and oromotor assessment, palpation of laryngeal structures, voice, and speech quality assessment and an observation of eating and drinking.

There are no agreed standards for long-term voice assessment, although review papers call for structured, standardized protocols, with measurements taken at baseline and long-term (27, 28). These typically include clinician rated scales, intelligibility rating, and acoustic measures (29). Examples of scales and measures used in HNC are given in Table 1.

**Table 1:** Examples of voice outcome measures used in HNC.

**Voice measures**	**Description**
Patient reported measures	Voice handicap index VHI-10 (30). 10 items on 5 point scale
	Voice-related quality of life V-RQOL (31). 10 items, 5 point scale and an overall score
	Voice symptom scale VoiSS (32). 30 items 3 subscales impairment-related, emotional, and physical symptoms
	Vocal performance questionnaire VPQ (33). 12 items, 5 point ordinal scale
Clinician rated measures	GRBAS (34) roughness, breathiness, asthenia, strain. 4 point scale for each area and overall grade
	Consensus Auditory-Perceptual Evaluation of Voice (CAPE V) (35). Overall severity, roughness, breathiness, strain, pitch, loudness. 6 vocal attributes measured on a visual analog scale
Acoustic and aerodynamic measures	Fundamental frequency**-**vocal fold vibration frequency
	Pertubation**-**Jitter: irregularities in vocal fold frequency, Shimmer: amplitude irregularities.
	Harmonic to noise ratio**-**ratio of sound frequencies to noise energy in the voice
	Maximum phonation time-glottic efficiency

### Patient Reported Outcome Measures

Measures of function in HNC should include patient-reported outcomes both pre- and post-treatment. Swallowing questionnaires and symptom report tools capture patients' perspectives of dysphagia, but they are only weakly correlated with swallowing impairment and aspiration, the relationship being particularly poor beyond 12 months post-treatment. They are therefore not interchangeable with clinical assessment (36, 37). The most commonly reported swallowing questionnaires used in HNC care include M.D. Anderson Dysphagia Inventory (38), SWAL-QOL (39), Sydney Swallow Questionnaire (40), and the EAT-10 (41). Examples of patient reported voice questionnaires include Voice Handicap Index (30), Voice Symptom Scale (32), and the Vocal Performance Questionnaire (42).

### Instrumental Assessment

Instrumental examination(s) is indicated for a thorough investigation of pathophysiology. Videofluoroscopy (VF) and Fiberoptic Endoscopic Evaluation of Swallowing (FEES®) are the most commonly used instrumental swallow assessments (43). VF is a recorded radiographic study of the swallowing structures, their movement and co-ordination. It is usually conducted in the lateral and anterior-posterior plane. Test boluses are mixed with radio-opaque material to enable visualization. FEES® allows a direct view of nasolaryngopharyngeal anatomy and physiology followed by an assessment of swallowing function (44, 45). A range of fluid and food can be given, without requiring radio opaque contrast. This may be combined with a voice assessment, which also requires a stroboscopic light source to fully assess vocal fold function and mucosal wave pattern.

The presentation of late treatment effects on instrumental assessment constitutes a number of features such as;

- Excessive external lymphedema, with underlying tissue hardening.
- Internal edema in critical structures and spaces for functioning e.g., true vocal folds, epiglottis, pyriform sinuses.
- Thickening of structures e.g., pharyngo-epiglottic folds creating a shelf-like barrier to bolus flow.
- Muscle thinning e.g., atrophied tongue base and pharyngeal wall.
- Palsies of the tongue, vocal fold, soft palate.
- Excessively dry laryngopharyngeal muscosa.

The pathophysiology, safety, and efficiency of swallowing can be analyzed and measured using rating scales, a selection of which are presented in Table 2.

**Table 2:** Examples of swallowing rating scales for FEES® and VF.

**Rating scale**	**Assessment**	
	FEES	VF
Penetration Aspiration Scale (49) 8-point scale describing laryngeal penetration and aspiration and sensory response	Yes	Yes
The MBS Impairment Scale (50) 16-item scale of swallow physiology and residue	No	Yes
Dynamic Image Grade of Swallowing Toxicity (DIGEST) (51) Summary score for swallow safety and efficiency	No	Yes
Oropharyngeal swallow efficiency (52) Combined measure of residue and aspiration	No	Yes
Patterson's Oedema Scale (53) 4-point scale of laryngopharyngeal structures and spaces	Yes	No
The Boston Residue and Clearance Scale (54) 11-point scale scoring amount, location and clearance of residue	Yes	No
Yale Pharyngeal Residue Severity Rating Scale (55), 5-point scale on amount of pharyngeal residue	Yes	No

## Monitoring

Cancer surveillance reviews are usually offered up to 5 years following treatment (1). Swallowing and voice function may not be routinely tested, unless patients report a problem. One preliminary study proposes four questions that could be used as part of a clinical assessment to detect dysphagia i.e., Do you have difficulties: (1) drinking? (2) eating? (3) swallowing? (4) Do you cough when eating/drinking? When three or more of these questions were answered affirmatory, the likelihood of aspiration observed on VF increased (46). Given afore described mortality risks, and the preponderance of sub-clinical aspiration, the immediate clinical challenge is identifying those at risk of developing pneumonia, using reliable, repeatable, and cost-effective tests.

### Identifying Patients at Risk of Aspiration Pneumonia

Healthy adults can experience trace aspiration without adverse consequences, and not every HNC survivor with aspiration appears to develop pneumonia. In a series of partial laryngectomy patients, 65% were identified with aspiration, but without x-ray evidence of pneumonia or infection (47). There may be additional factors to be taken into account, when weighing up pneumonia risk. For example, older adults in a care facility developed pneumonia only when aspiration co-occurred with poor oral hygiene, dependence on others for eating, poor dentition and co-morbidities (48). Most HNC studies have explored patient and disease characteristics and treatment type as predictors of pneumonia, with no clear pattern emerging (see Table 3). Further work on identifying predictor variables for aspiration pneumonia to be used as part of an algorithm for monitoring purposes is required.

**Table 3:** HNC studies reporting predictors of (1) aspiration pneumonia^*^ (2) aspiration pneumonia-related death^**^ ≠ Wang et al. (24) only included patients with a diagnosis of aspiration pneumonia.

**Author**	**N**	**T site**	**Tx**	**% AP**	**Predictors of AP**	**Non-predictive**
Hunter et al. (69)	72	OPSCC	C-IMRT	22%	T-stage, patient report, aspiration on VF^*^	CTCAE
Xu et al. (20)	3513	Mixed	Surgery and RT, CRT, RT	16% 1Y 24% 5Y	Hypopharynx or NPC, gender, age, co-morbidity, primary RT, care at non-teaching hospital^*^	Surgery, stage, type of CT, type of RT
Kawai et al. (70)	305	Mixed	CRT	21%	Alcohol, sleeping pills, oral hygiene, hypoalbumemia, presence of other cancer^*^	
Madan et al. (71)	85	Mixed	Surgery & RT, CRT, RT	60%	Pharynx cancer, T stage^**^	Age, gender, tx modality, smoking, co-morbidity, treatment intent, baseline swallowing
Wang et al. (24)	113	NPC	RT +/– CT IMRT	100% ≠	age, smoking, weight loss, lower CN palsy^**^	
O'Hare et al. (72)	206	Mixed	RT +/– CT	15%	Larynx cancer, dose to cricopharynx^**^	Gender, chemotherapy

*NPC, nasopharynx cancer; OPSCC, oropharynx cancer; T, tumor; Tx, treatment; CRT, chemoradiotherapy; RT, radiotherapy; CT, chemotherapy*.

### Screening Assessments

Instrumental assessments provide a more objective way of measuring swallowing, but have limited access and high cost, due to the need for specialist equipment and personnel (56). In Stroke care, early detection of dysphagia by screening reduces pulmonary complications, length of hospital stay, and overall health care costs (57). No such programme exists for HNC survivors, but the following section reviews candidate screening tests for this group.

#### Water Swallow Test

A water swallow test (WST) is the most commonly reported aspiration screening tool (58). Two systematic reviews report a range of protocols, predominantly for testing neurological dysphagia (56, 58–61). Using prescribed amounts of water and recording coughing or a wet voice gave the most accurate results for identifying aspiration (56, 62). Consecutive sips with large volumes had the best sensitivity (91% CI 89, 93%) while, single sips of water were better at ruling out aspiration (90% CI 86, 93%) (58). We have reported on the timed 100 mLs WST in HNC, recording swallowing performance overtime, as well as a screening test for aspiration (63). This test was acceptable for identifying aspiration in early post-operative or chemoradiotherapy patient groups (63, 64), but its reliability for late effects patients has not been investigated.

#### Cough Reflex Test

This test assesses the cough reflex by introducing a tussive agent such as citric acid via a facemask and nebulizer and observing for a responsive cough (65). The outcome is judged on reflexive cough strength and has been used as screening test in Stroke (65), Parkinson's Disease (66), and post-extubation (67). The absence of a cough reflex in neurological patients has a sensitivity of 69% (95% CI 55, 81%) and specificity of 71% (95% CI 63, 77%) to detect aspiration (65). No studies have reported on its use in HNC, but given that late effects can result in silent aspiration, further investigation is warranted.

### Pulmonary Function

Many patients elect to eat and drink despite experiencing aspiration. Assessments to monitor pulmonary function such as spirometry and flow-volume loops may be indicated together with close collaboration with Respiratory Physicians to develop mechanisms to detect deterioration in chest status.

## Future Directions

There are a number of current trials investigating treatment modulation to prevent or reduce toxicities without compromising survival (68). In the meantime, further work on late functional effects is indicated in the following areas.

- Information on the long-term outcomes for voice and speech.
- Identification of predictors of significant fibrosis and exploration of preventative interventions.
- Identification of predictor variables for pneumonia in patients who aspirate.
- Reliable screening tests for identifying aspiration and dysphagia for long-term follow up.
- Accurate patient information regarding late treatment effects.
- Clinical care pathways for identifying and monitoring late functional effects.

## Conclusion

The number of HNC survivors is rising and this trend is set to continue. Late functional effects of treatment is a common, distressing, and potentially life-threatening problem. Assessment and monitoring of HNC survivors in the long-term is an important step for identifying those at risk, as early as possible. Reliable, acceptable, and repeatable screening assessments are needed to address this growing problem.

## Author Contributions

The author confirms being the sole contributor of this work and has approved it for publication.

### Conflict of Interest Statement

The author declares that the research was conducted in the absence of any commercial or financial relationships that could be construed as a potential conflict of interest.
